# Improved glucose tolerance in acyl CoA:diacylglycerol acyltransferase 1-null mice is dependent on diet

**DOI:** 10.1186/1476-511X-6-2

**Published:** 2007-01-19

**Authors:** Steven JY Wang, Claire Cornick, Jacqueline O'Dowd, Michael A Cawthorne, Jonathan RS Arch

**Affiliations:** 1 Clore Laboratory, University of Buckingham, Buckingham MK18 1EG, UK

## Abstract

**Background:**

Mice that lack acyl CoA:diacylglycerol acyltransferase (*Dgat1*^-/- ^mice) are reported to have a reduced body fat content and improved glucose tolerance and insulin sensitivity. Studies so far have focussed on male null mice fed a high fat diet and there are few data on heterozygotes. We compared male and female *Dgat1*^-/-^, *Dgat1*^+/- ^and *Dgat1*^+/+ ^C57Bl/6 mice fed on either standard chow or a high fat diet.

**Results:**

Body fat content was lower in the *Dgat1*^-/- ^than the *Dgat1*^+/+ ^mice in both experiments; lean body mass was higher in male *Dgat1*^-/- ^than *Dgat1*^+/+ ^mice fed on the high fat diet. Energy intake and expenditure were higher in male *Dgat1*^-/- ^than *Dgat1*^+/+ ^mice; these differences were less marked or absent in females. The body fat content of female *Dgat1*^+/- ^mice was intermediate between that of *Dgat1*^-/- ^and *Dgat1*^+/+ ^mice, whereas male Dgat1^+/- ^mice were similar to or fatter than *Dgat1*^+/+ ^mice. Glucose tolerance was improved and plasma insulin reduced in *Dgat1*^-/- ^mice fed on the high fat diet, but not on the chow diet. Both male and female *Dgat1*^+/- ^mice had similar glucose tolerance to *Dgat1*^+/+ ^mice.

**Conclusion:**

These results suggest that although ablation of DGAT1 improves glucose tolerance by preventing obesity in mice fed on a high fat diet, it does not improve glucose tolerance in mice fed on a low fat diet.

## Background

The final step of triglyceride synthesis in mammals is catalysed by the enzymes acyl CoA:diacylglycerol acyltransferase 1 and 2 (DGAT1 and DGAT2), which have dissimilar amino acid sequences. Both enzymes are widely expressed and are highly expressed in adipose tissue and liver [1]. Previous reports have described resistance to diet-induced obesity [2] and amelioration of obesity due to the *A*^*y*^, but not the *Lep*^*ob *^or *LepR*^*db*^, mutation [3] in DGAT1-deficient (*Dgat1*^-/-^) mice. Resistance to obesity was associated with increased reductions in body weight and food intake in response to peripherally but not centrally administered leptin [3,4]. Food intake relative to body weight was either unchanged [2] or raised [3,4], and energy expenditure relative to body weight was raised [2], suggesting that the primary effect of DGAT1 deficiency is on the energy expenditure side of the energy balance equation. Increased locomotor activity and brown adipose tissue thermogenesis have been invoked to account for increased energy expenditure [2,3,5]. It appears, however, that fat oxidation is increased in liver as well as skeletal muscle and brown adipose tissue because the concentration of diacylglycerol was reduced in the liver of *Dgat1*^-/- ^mice [3]. This finding is paradoxical since one might expect the concentration of diacylglycerol – the substrate of DGAT1 – to be raised in *Dgat1*^-/- ^mice, but it can be rationalised if fat oxidation is somehow increased.

Stimulation of fatty acid oxidation is usually associated with improved insulin sensitivity and glucose tolerance, possibly because the concentrations of lipid metabolites that inhibit the insulin signalling pathway (e.g. long chain fatty acyl CoA, diacylglycerol, ceramide) are reduced [6]. Moreover, in the longer term, insulin sensitivity will improve due to the reduced plasma non-esterified fatty acid concentration and altered adipokine profile associated with decreased adipocyte lipid stores. Therefore it is not surprising that *Dgat1*^-/- ^C57Bl/6 mice have been reported to show improved insulin sensitivity [3,7,8]. However, overexpression of DGAT1 in adipose tissue of C57Bl/6 mice was associated with obesity but not with impaired glucose disposal [9]. By contrast, overexpression of DGAT1 in adipose tissue of FVB mice, a strain known to be resistant to diet-induced obesity, was not associated with obesity, but was associated with insulin resistance [10]. These results support the view that an increased capacity for triacylglycerol synthesis is not detrimental to insulin sensitivity provided it is confined to adipocytes and newly formed adipocytes can accommodate any excess triacylglycerol.

We found in a pilot experiment [11] that, in contrast to a previous report [2] glucose tolerance and insulin tolerance were actually worse, and fasting plasma insulin was raised in *Dgat1*^-/- ^compared to wildtype mice fed on a chow (low fat) diet. The knockout mice appeared to border on a state of 'functional lipodystrophy'. The wildtype mice used in this pilot experiment were age-matched to the *Dgat1*^-/- ^mice rather than being littermates, raising the possibility that a minor variation in genetic background could have been responsible for the difference. We have therefore now compared *Dgat1*^-/- ^and littermate *Dgat1*^+/+ ^mice fed on both chow and high fat diets with respect to glucose homeostasis and energy balance. While we do not repeat the finding of impaired glucose tolerance, we do find that it is only on the high fat diet that glucose tolerance is improved in the *Dgat1*^-/- ^mice. Since there are limited data on the phenotype of heterozygote (*Dgat1*^+/-^) mice [1], these were included in the study to give some perspective on how much inhibition of DGAT1 might be required to treat obesity and type 2 diabetes. We also compared the effects of knockout of the *Dgat1 *gene in both male and female mice, whereas previous studies have focussed on males.

## Results

**Figure 1:**
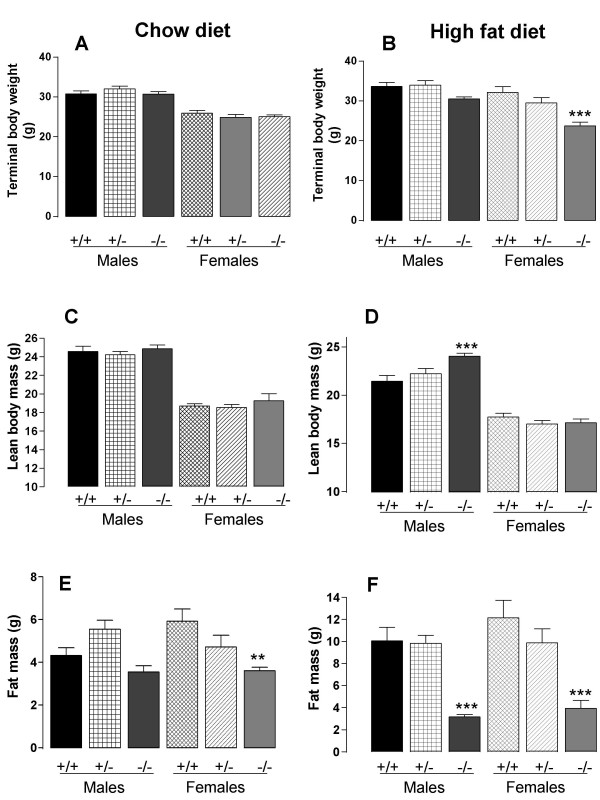
Final body weight (A, B), lean body mass (C, D) and fat mass (E, F) in *Dgat1*^+/+ ^(+/+), *Dgat1*^+/- ^(+/-) and *Dgat1*^-/-^(-/-) mice fed on a chow diet (A, C, E) or a high fat diet (B, D, F). Results are means of 12 values ± S.E., except that there were 6 female *Dgat1*^-/- ^mice fed on the high fat diet. **P < 0.01; ***P < 0.001, compared to *Dgat1*^+/+ ^mice of the same sex.

When the mice were fed throughout on a chow diet, terminal body weight and lean body mass (at 20 weeks of age) were not significantly affected by genotype (Figs. 1A, C). However, the female *Dgat1*^-/- ^mice had a higher body weight than either the *Dgat1*^+/- ^or the *Dgat1*^+/+ ^mice at 9 weeks of age (21.5 ± 0.3, 20.0 ± 0.4, 19.3 ± 0.3 g respectively; n = 12; *P *< 0.05). When the mice were fed on the high fat diet, the male *Dgat1*^-/- ^mice had a markedly higher lean body mass compared to the *Dgat1*^+/+ ^mice (Fig. 1D), but this was not reflected in terminal body weight (Fig. 1B) because body fat content was reduced (Fig. 1F).

This reduction in body fat content in *Dgat1*^-/- ^mice when the mice were fed on a high fat diet was larger (7 g in males and 8 g in females, compared to *Dgat1*^+/+ ^mice; Fig. 1F) than when the mice were fed on chow (2 g in females, and 0.7 g, which was not statistically significant, in males; Fig. 1E). The *Dgat1*^+/- ^mice did not have significantly altered body fat content compared to the *Dgat1*^+/+ ^mice on either diet, but in females their body fat content was intermediate between that of the *Dgat1*^+/+ ^and *Dgat1*^-/- ^mice, whereas in males the *Dgat1*^+/- ^mice fed on the chow diet actually tended to have a higher body fat content than the *Dgat1*^+/+ ^mice (*P *= 0.046 by Fisher least significant difference test, but not significant after Bonferroni correction for four tests). Adiposity was reflected in terminal fat pad weights and plasma leptin levels (Table 1). The male *Dgat1*^+/- ^mice had significantly higher fat pad weights and leptin levels than *Dgat1*^+/+ ^mice even after Bonferroni correction.

**Table 1:** Terminal plasma leptin and perigenital fat pad weights

	Males			Females		
	+/+	+/-	-/-	+/+	+/-	-/-

Chow diet:						
Leptin (ng/ml)	2.2 ± 1.8	4.4 ± 1.8***	1.5 ± 0.4	5.5 ± 3.7	4.2 ± 2.4	1.9 ± 0.2
Fat pad weight (g)	0.50 ± 0.07	0.78 ± 0.11*	0.32 ± 0.03***	0.70 ± 0.08	0.56 ± 0.08	0.31 ± 0.05***
High fat diet:						
Leptin (ng/ml)	16.3 ± 2.1	16.0 ± 2.4	1.2 ± 0.3***	21.1 ± 1.5	19.6 ± 2.2	2.8 ± 0.3***
Fat pad weight (g)	1.57 ± 0.13	1.52 ± 0.7	0.32 ± 0.04***	1.67 ± 0.18	1.31 ± 0.15	0.48 ± 0.14***

Values are means ± S.E. of 10 to 12 values, except that there were 5 values for plasma leptin and 6 for fat pad weight for the female Dgat1^-/- ^mice fed on the high fat diet.

* *P *< 0.05; *** *P *< 0.001 compared to wildtype mice of the same sex.

**Figure 2:**
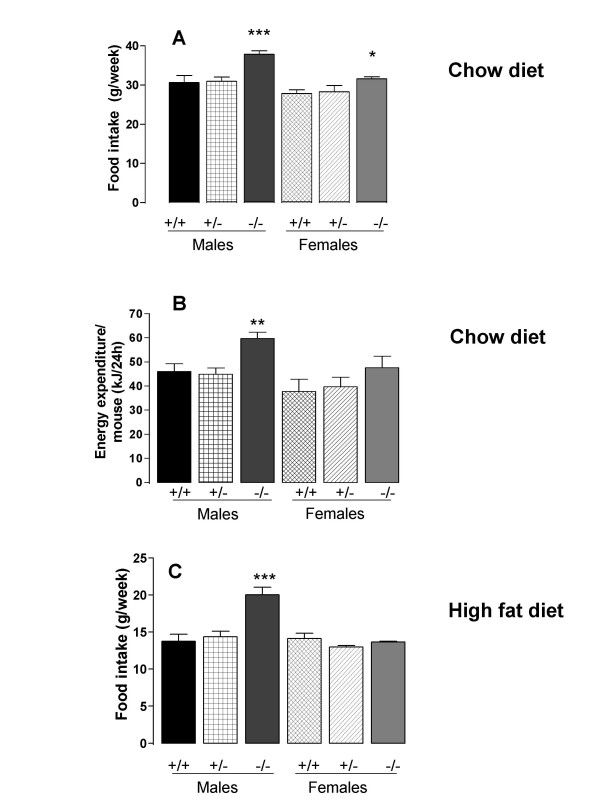
Food intake (A) and energy expenditure (B) in *Dgat1*^+/+ ^(+/+), *Dgat1*^+/- ^(+/-) and *Dgat1*^-/-^(-/-) mice fed on a chow diet, and food intake (C) in mice fed on a high fat diet. Results are means of 4 values ± S.E., except that there were two values for food intake (C) for the female *Dgat1*^-/- ^mice fed on the high fat diet (and hence the top of the error bar corresponds to the higher of these two values, which differed by only 1.6%). *P < 0.05; **P < 0.01; ***P < 0.001 compared to *Dgat1*^+/+ ^mice of the same sex.

In the chow diet experiment, food intake from 9 to 20 weeks of age was higher in both male and female *Dgat1*^-/- ^than in *Dgat1*^+/+ ^mice (Fig. 2A). Energy expenditure at 12 weeks of age was raised in the male *Dgat1*^-/- ^mice (Fig. 2B). Energy expenditure was not significantly raised in the female *Dgat1*^-/- ^mice, but there was a trend (*P *= 0.087 by Fisher test) to an increase that paralleled the food intake data. In the high fat diet experiment, food intake from 12 to 30 weeks of age was higher in male *Dgat1*^-/- ^than *Dgat1*^+/+ ^mice, but there was no difference in food intake between the female genotypes (Fig. 2C). Food intake and energy expenditure in the *Dgat1*^+/- ^mice were no different from in the *Dgat1*^+/+ ^mice.

**Figure 3:**
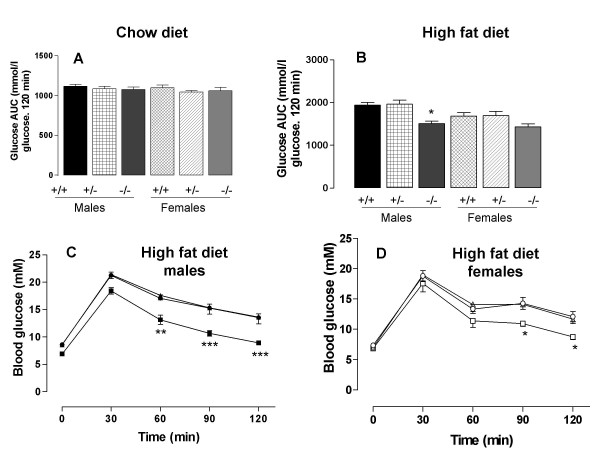
Total area under the glucose tolerance curve in *Dgat1*^+/+ ^(+/+), *Dgat1*^+/- ^(+/-) and *Dgat1*^-/-^(-/-) mice fed on a chow diet (A) or a high fat diet (B); and glucose tolerance curves for male (C) and female (D) mice fed on the high fat diet. Symbols in C and D: *Dgat1*^+/+^, ● ○; *Dgat1*^+/-^, ▲ △; *Dgat1*^-/-^, □ ■. Results are means of 12 values, except that there were 6 female *Dgat1*^-/- ^mice fed on the high fat diet. *P < 0.05; **P < 0.01; ***P < 0.001 compared to *Dgat1*^+/+ ^mice of the same sex.

**Figure 4:**
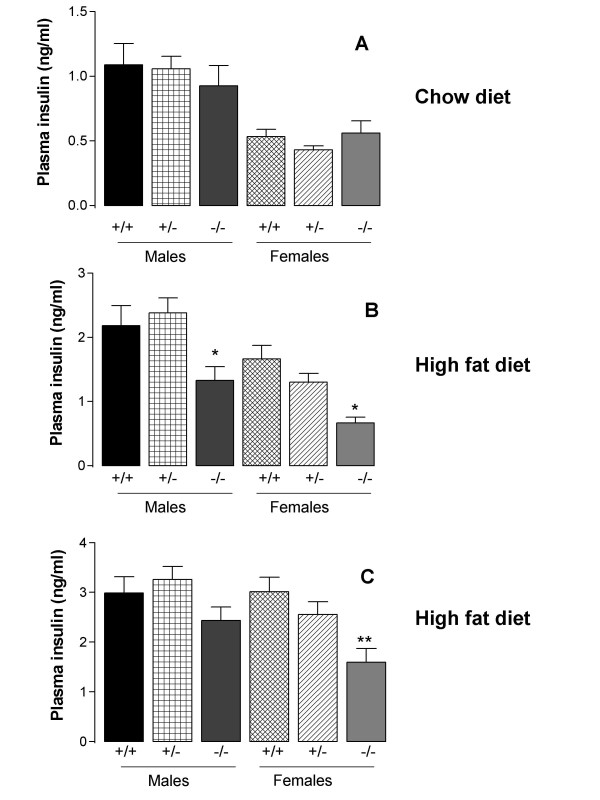
Plasma insulin concentrations in *Dgat1*^+/+ ^(+/+), *Dgat1*^+/- ^(+/-) and *Dgat1*^-/-^(-/-) mice fed on a chow diet (A) or a high fat diet (B) immediately before administration of glucose in the glucose tolerance tests, and also 30 min after administration of glucose in the high fat-fed mice (C). Results are means of 12 values, except that there were 6 female *Dgat1*^-/- ^mice fed on the high fat diet. * *P *< 0.05; ** *P *< 0.01; *** *P *< 0.001 compared to *Dgat1*^+/+ ^mice of the same sex.

An unexpected finding was that in the chow diet experiment, glucose tolerance was, by contrast with a previous report [2], very similar in mice of the three different genotypes (Fig. 3A). However, in the high fat diet experiment, the area under the glucose tolerance curve was, as expected, lower in the male *Dgat1*^-/- ^than in the male *Dgat1*^+/- ^or *Dgat1*^+/+ ^mice, and in both male and female *Dgat1*^-/- ^mice the blood glucose concentration was reduced at the later time-points (Figs. 3C, D). The fasting plasma insulin concentration was similarly unaffected by genotype in mice fed on the chow diet (Fig. 4A), but lower in the *Dgat1*^-/- ^than the *Dgat1*^+/- ^or *Dgat1*^+/+ ^mice fed on the high fat diet (Fig. 4B). The plasma insulin concentration was also lower in the female *Dgat1*^-/- ^than the *Dgat1*^+/- ^or *Dgat1*^+/+ ^mice 30 min after the glucose load (Fig. 4C).

## Discussion

Our results are consistent with a previous report [2] in showing that *Dgat1*^-/- ^mice have a lower body fat content than *Dgat1*^+/+ ^mice and are resistant to diet-induced obesity. They also agree that energy turnover is increased in male *Dgat1*^-/- ^mice, although this was less evident in female *Dgat1*^-/- ^mice. They differ, however, from previous reports that show [2] or imply [7] that glucose tolerance is improved in *Dgat1*^-/- ^mice fed on a chow diet.

Body fat content was higher in the *Dgat1*^+/+ ^mice fed on the high fat diet compared with the same mice fed on the chow diet, but it was similar in *Dgat1*^-/- ^mice fed on the high fat and chow diets. Consequently, the effect of ablation of the gene was greater in mice fed on the high fat diet. The effect of ablation of the *Dgat1 *gene was as apparent in female as in male mice, which have been the focus of previous studies.

Why the absence of an enzyme involved in triacylglycerol synthesis should increase fat oxidation and energy expenditure is not known. One study suggested that a factor released in increased amounts from the adipose tissue of *Dgat1*^-/- ^mice could play a role [7], but it seems unlikely that this factor is adiponectin [1,12]. Plasma adiponectin was decreased in male *Dgat1*^-/- ^mice in our pilot experiment [11], which is also inconsistent with this hypothesis. Increased locomotor activity in *Dgat1*^-/- ^mice raises the possibility that ablation of DGAT1 in the brain raises energy expenditure in the periphery. Thus hypothalamic lipid metabolites are known to affect energy expenditure [13]. However, overexpression of DGAT1 in adipose tissue alters body composition [9,10], and overexpression of DGAT1 in rat isolated islets of Langerhans increases triglyceride synthesis [14]. Therefore, at least in adipose tissue and islets, DGAT1 can have a direct effect on triglyceride storage.

An unexpected finding that may in part account for increased energy expenditure in male *Dgat1*^-/- ^mice was that lean body mass, which has more influence on energy expenditure than fat mass [15-17], was increased in male *Dgat1*^-/- ^mice fed on the high fat diet. Lean body mass was not raised significantly in the particular group – male *Dgat1*^-/- ^mice – where energy expenditure was significantly raised in the present study, but energy expenditure was measured at 12 weeks of age and body composition at 20 weeks of age. Other workers have not observed increased lean body mass in *Dgat1*^-/- ^mice [12], although a trend to increased percentage protein content in *Dgat1*^-/- ^mice fed on a chow diet has been reported [2].

Our finding that glucose tolerance was not improved in *Dgat1*^-/- ^mice fed on a chow diet compares with studies on the overexpression of DGAT1. This did not result in impaired glucose disposal in a strain of mice that was able to respond with increased adipose tissue fat stores [18], but did cause insulin resistance in a strain that was unable to accommodate extra lipid in adipocytes [10]. In the current study, in contrast to our pilot experiment [11], ablation of DGAT1 did not impair glucose tolerance, but nevertheless, inhibition of lipid synthesis in animals that have a low capacity for adipocyte triglyceride accumulation may risk exacerbating insulin sensitivity. In this regard, it is interesting that lipodystrophic animals and humans are characterised by insulin resistance [19] and a recent report suggests that lipodystrophic humans have an increased lean body mass [20], a feature displayed by male *Dgat1*^-/- ^mice in the current work.

There were no significant differences between the heterozygote (*Dgat1*^+/-^) and *Dgat1*^+/+ ^mice, except for the surprising finding of increased fat pad weights and leptin concentration in the male *Dgat1*^+/- ^mice fed on chow. This contrasts with a previous report that male heterozygote mice had a total fat pad weight intermediate between *Dgat1*^-/- ^and *Dgat1*^+/+ ^mice [1]. Female heterozygote mice, by contrast, did have an intermediate phenotype in terms of fat mass (Fig. 2). Perhaps compensatory mechanisms, such as increased DGAT2 activity, were more pronounced in males than in females. Glucose tolerance was very similar in heterozygote and wildtype mice. This suggests that to treat type 2 diabetes with an inhibitor of DGAT1 it may be necessary to inhibit activity markedly. The activities of DGAT1 in the wildtype and heterozygote mice were not compared, however.

## Conclusion

*Dgat1*^-/- ^mice had a lower body fat content than wildtype mice and males had an increased lean body mass when they were fed on a high fat diet. Improved glucose tolerance and reduced plasma insulin levels were apparent only when the mice were fed on a high fat diet. Inhibition of triglyceride synthesis does not improve glucose tolerance if adipocyte lipid stores are already low.

## Methods

### Animals

Procedures were conducted in accordance with the University of Buckingham Home Office UK project licence under the Animals (Scientific Procedures) Act (1986) and as agreed by the University of Buckingham Ethical Review Board.

Three male *Dgat1*^-/- ^and three female *Dgat1*^+/- ^mice back-crossed onto a C57Bl/6 background for ten generations (B6.129S4 – *Dgat1*^*tm*1*Far*^/J) were purchased from Jackson Laboratories (Bar Harbor, Maine, USA) and a colony was expanded in our facilities, breeding from male *Dgat1*^-/- ^and female *Dgat1*^+/- ^mice. The method used to delete the *Dgat1 *gene has been reported previously [2]. The offspring from the breeding of the *Dgat1*^-/- ^and *Dgat1*^+/- ^mice were genotyped between 4 to 5 weeks of age and in order to generate littermates of all three possible genotypes, male and female *Dgat1*^+/- ^mice were used for further rounds of breeding to obtain the experimental mice. Genomic DNA was extracted from tail tip samples and *Dgat1 *and *neomycin *genes detected by PCR using the primers previously described [2]. The presence of both *neomycin *and *Dgat1 *genes indicated heterozygosity.

### Protocol

The mice were fed on a standard chow diet that contained 10% fat, 70% carbohydrate and 20% protein by energy (14.0 kJ/g metabolisable energy; Beekay Feed, B&K Universal Ltd., Hull, UK), or they were fed on chow until they were 12 weeks old and then on a sweetened high fat diet that contained by energy 45% fat, 35% carbohydrate (of which half was sucrose) and 20% protein (19.3 kJ/g metabolisable energy; diet code D12451; Research Diets, New Brunswick, USA). They were housed at 22°C with lights on from 07.00 to 19.00 h.

There were twelve mice of each sex and genotype (*Dgat1*^+/+^, *Dgat1*^+/- ^and *Dgat1*^-/-^) housed in threes, except that only six female *Dgat1*^-/- ^mice were fed on the high fat diet. Food intake for each cage of mice was measured daily and body weight was measured weekly from the age of 9 or 13 weeks in the chow or high fat diet experiments, respectively. Energy expenditure was measured at 12 weeks of age in mice fed on the chow diet. Glucose tolerance tests were performed at 15 and 24 weeks of age in the chow and high fat diet experiments respectively. The mice were killed after a 5-hour fast aged 20 (chow diet) or 30 (high fat diet) weeks and blood was taken for the measurement of plasma leptin (Crystal Chem Inc, Chicago, IL). Body composition was determined by dual energy X-ray absorptiometry (Lunar PIXImus 2 mouse densitometer and version 1.46 software; GE Medical, Bedford, UK).

### Glucose tolerance

Mice were fasted for 5 h from 09.00 h before administration of glucose (2 g/kg, i.p. body weight). Blood samples were taken from the tip of the tail after the topical application of a local anaesthetic (lignocaine gel) at 30-minute intervals. Glucose and insulin were measured as described previously [21].

### Energy expenditure

Energy expenditure was measured over 24 h, beginning at 10.00 h in the mice's home cage by open circuit indirect calorimetry using the equation of Weir [22]. The volume of the mouse cages was 23 litres and the flow rate was 0.8 l/min. Such a system has a calculated half-life of 23.5 min for responding to a step change in energy expenditure [17]. It is therefore not suitable for instant measurement of energy expenditure but with the mice undisturbed in their home cages, it is ideal for measurement of daily energy expenditure. The energy expenditure of all the male mice of all the different genotypes was measured in a single run over 24 h and after recalibration, the energy expenditure of all the female mice was measured on the following day.

### Data analysis

Results are presented as means ± S.E. They were analysed by one-way analysis of variance followed by Bonferroni's post-test for selected comparisons (wild-type mice with heterozygous or homozygous mutant mice of the same sex) unless stated otherwise.

## Authors' contributions and conflict of interest

JA supervised all stages of the study. SW planned and conducted the experiments with support in some experiments from CC and JO. SW analysed and interpreted the data, and supported JA in the writing of the manuscript. MC provided advice at all stages. SW is now an employee of AstraZeneca, who have an interest in DGAT-1 inhibitors.

## References

[B1] Chen HC, Farese RV (2005). Inhibition of triglyceride synthesis as a treatment strategy for obesity: lessons from DGAT1-deficient mice. Arterioscler Thromb Vasc Biol.

[B2] Smith SJ, Cases S, Jensen DR, Chen HC, Sande E, Tow B, Sanan DA, Raber J, Eckel RH, Farese RV (2000). Obesity resistance and multiple mechanisms of triglyceride synthesis in mice lacking Dgat. Nat Genet.

[B3] Chen HC, Smith SJ, Ladha Z, Jensen DR, Ferreira LD, Pulawa LK, McGuire JG, Pitas RE, Eckel RH, Farese RV (2002). Increased insulin and leptin sensitivity in mice lacking acyl CoA:diacylglycerol acyltransferase 1. J Clin Invest.

[B4] Chen HC, Ladha Z, Farese RV (2002). Deficiency of acyl coenzyme a:diacylglycerol acyltransferase 1 increases leptin sensitivity in murine obesity models. Endocrinology.

[B5] Chen HC, Ladha Z, Smith SJ, Farese RV (2003). Analysis of energy expenditure at different ambient temperatures in mice lacking DGAT1. Am J Physiol Endocrinol Metab.

[B6] Clapham JC, Arch JR (2006). Thermogenic and metabolic antiobesity drugs: rationale and opportunities. Diabetes Obesity and Metabolism.

[B7] Chen HC, Jensen DR, Myers HM, Eckel RH, Farese RV (2003). Obesity resistance and enhanced glucose metabolism in mice transplanted with white adipose tissue lacking acyl CoA:diacylglycerol acyltransferase 1. J Clin Invest.

[B8] Chen HC, Rao M, Sajan MP, Standaert M, Kanoh Y, Miura A, Farese RV, Farese RV (2004). Role of adipocyte-derived factors in enhancing insulin signaling in skeletal muscle and white adipose tissue of mice lacking Acyl CoA:diacylglycerol acyltransferase 1. Diabetes.

[B9] Chen HC, Stone SJ, Zhou P, Buhman KK, Farese RV (2002). Dissociation of obesity and impaired glucose disposal in mice overexpressing acyl coenzyme a:diacylglycerol acyltransferase 1 in white adipose tissue. Diabetes.

[B10] Chen N, Liu L, Zhang Y, Ginsberg HN, Yu YH (2005). Whole-body insulin resistance in the absence of obesity in FVB mice with overexpression of Dgat1 in adipose tissue. Diabetes.

[B11] Wang SJY, Arch JR (2004). Reduced insulin sensitivity and glucose tolerance in DGAT-1 knockout mice. Int J Obes.

[B12] Streeper RS, Koliwad SK, Villanueva CJ, Farese RV (2006). Effects of DGAT1 deficiency on energy and glucose metabolism are independent of adiponectin. Am J Physiol Endocrinol Metab.

[B13] Wolfgang MJ, Lane MD (2006). Control of energy homeostasis: role of enzymes and intermediates of fatty acid metabolism in the central nervous system. Annu Rev Nutr.

[B14] Kelpe CL, Johnson LM, Poitout V (2002). Increasing triglyceride synthesis inhibits glucose-induced insulin secretion in isolated rat islets of langerhans: a study using adenoviral expression of diacylglycerol acyltransferase. Endocrinology.

[B15] Selman C, Lumsden S, Bunger L, Hill WG, Speakman JR (2001). Resting metabolic rate and morphology in mice (Mus musculus) selected for high and low food intake. J Exp Biol.

[B16] Speakman JR, Johnson MS, Heldmaier G and Klingenspor M (2000). Relationships between resting metabolic rate and morphology in lactating mice:what tissues are the major contributors to resting metabolism?. Life in the cold.

[B17] Arch JR, Hislop D, Wang SJ, Speakman JR (2006). Some mathematical and technical issues in the measurement and interpretation of open-circuit indirect calorimetry in small animals. Int J Obes (Lond).

[B18] Reitman ML, Arioglu E, Gavrilova O, Taylor SI (2000). Lipoatrophy revisited. Trends Endocrinol Metab.

[B19] Savage DB, Murgatroyd PR, Chatterjee VK, O'Rahilly S (2005). Energy expenditure and adaptive responses to an acute hypercaloric fat load in humans with lipodystrophy. J Clin Endocrinol Metab.

[B20] Wargent E, Sennitt MV, Stocker C, Mayes AE, Brown L, O'Dowd J, Wang S, Einerhand AW, Mohede I, Arch JR, Cawthorne MA (2005). Prolonged treatment of genetically obese mice with conjugated linoleic acid improves glucose tolerance and lowers plasma insulin concentration: possible involvement of PPAR activation. Lipids Health Dis.

[B21] Weir JB (1949). New methods for calculating metabolic rate with special reference to protein metabolism. J Physiol.

